# Adjuvant interferon alfa for melanoma: new evidence-based treatment recommendations?

**DOI:** 10.3747/co.v16i3.447

**Published:** 2009-05

**Authors:** A. Hauschild

**Keywords:** Melanoma, adjuvant therapy, interferon alfa

Malignant melanoma of the skin is the most frequent cause of mortality in skin cancer. Despite various efforts toward increased early detection and prevention of melanoma, approximately 20% of patients will die because of disseminated metastases. Once melanoma has spread to regional lymph nodes, survival diminishes to approximately 30%–55% depending on micro- or macrometastases and the number of involved nodes. It is well established that the tumour load within the lymph node or nodes is an important predictor for disease-free survival (dfs) and overall survival (os). Median survival for stage iv melanoma patients ranges from 6 months to 12 months, and to date, fewer than 10% of all stage iv patients demonstrate long-term survival. The poor outcome for patients with advanced metastatic melanoma indicates an urgent need for effective adjuvant treatment modalities for patients with high-risk disease.

Since the 1970s, dozens of prospective randomized controlled clinical trials for intermediate- and high-risk melanoma patients have been performed. In contrast to the situation with other solid malignancies such as breast and colorectal cancer, no global consensus has been reached on an effective adjuvant treatment for melanoma patients^1^.

Approaches using unspecific immunostimulation (bacille Calmette–Guérin, among others), interferon γ, conventional chemotherapy, and vaccination have not been demonstrated to improve dfs or particularly os in comparison with untreated controls^2^.

Therefore, even in the year 2009, interferon alfa is the only agent being used in adjuvant treatment for melanoma that has showed at least a positive effect on relapse-free survival (rfs)—and in the case of high-dose interferon alfa (hdi), also a positive effect on os. However, those benefits provide only a modest improvement of rfs in the range of 5%–10% and os in the range of 2%–5%. However, the data are robust, given that the results of a meta-analysis also demonstrated statistically significant benefits for interferon-treated as compared with untreated patients^3^.

## Why Did These Study Results Not Lead to a Global Consensus About Interferon Treatment as a Standard of Care?

First, apart from a single clinical trial reported by Kirkwood *et al.,* which compared patients receiving hdi 2b with untreated controls [Eastern Cooperative Oncology Group (ecog) 1684], no other clinical trial has demonstrated a clear statistically significant benefit in os^4^. The question therefore arises whether a benefit in rfs is also a clinically meaningful endpoint. The quality-of-life-adjusted survival analysis for patients treated with hdi 2b in the United States revealed that patients are willing to accept significant toxicity for even a modest improvement of rfs in the absence of os benefit^5^. In the absence of good treatment alternatives apart from a “wait and see” policy, many German patients have also been willing to accept moderate-to-severe toxicity for minimal benefit when they are appropriately informed before treatment initiation. However, more attention should be paid to this clinically important question that affects the quality of life of melanoma patients so much.

Second, as already mentioned, the toxicity of the approved hdi 2b is significant but manageable^6^ Over the years of experience with this new drug regimen (approved in 1996 in the United States and Canada, and also later in Europe), the number of patients with toxicity-related treatment discontinuations has dropped significantly. In centres with many treated patients, the rate of treatment cessation is consistently in a range of 10% when established rules for a two-step interferon dose reduction scheme are respected^4^,^6^. The hdi scheme has already been implemented into national guidelines for the treatment of stage iii melanoma patients^7^,^8^; however, because of dose-limiting toxicity and, most likely, also because of the high cost of this treatment, hdi is outside the routine spectrum of adjuvant treatment modalities in some countries such as the Netherlands and the United Kingdom.

In Europe, the question of the optimal dose of interferon alfa is even more critical, because not only hdi, but also low-dose interferon alfa (ldi) 2a has been approved for the adjuvant treatment of melanoma. Interferon alfa 2a is approved for clinically lymph node–negative melanoma patients with a tumour thickness of at least 1.5 mm, which illustrates that not only patients who were sentinel node–negative, but also those who, before the introduction of sentinel lymph node dissection, were sentinel node–positive, have been treated. In the French multicentre melanoma trial conducted by Grob *et al.,* 499 stage ii melanoma patients with no clinical evidence of lymph node involvement received 3×10^6^ IU thrice weekly for 18 months^9^. Because of the moderate toxicity and lower costs as compared with hdi, ldi became a standard of care in many European countries (that is, Germany, Austria, Switzerland, France). However, in countries such as the Netherlands and the United Kingdom, neither the positive effect of ldi on dfs, nor its favourable toxicity profile has convinced physicians to use this scheme.

An illustrative example of the complex situation that results comes from my own melanoma centre in Kiel, Germany. Danish melanoma patients who want to receive adjuvant interferon outside of a clinical trial are treated there because interferon is not a standard of care in Denmark. “Melanoma tourism” to neighbouring centres in Germany is a logical consequence of that policy. To sum up, neither in Europe nor in the United States or Canada has a consensus been reached on the best treatment schedule or even use of interferon alfa at all^10^.

In a large recently published clinical trial conducted by Eggermont *et al.*^11^ for the European Organization for the Research and Treatment of Cancer (eortc) Melanoma Group, patients receiving pegylated interferon alfa 2b showed improved rfs for fully resected stage iii melanoma with micro-or macrometastases. The trial compared patients treated using pegylated interferon alfa 2b with untreated controls. The difference in rfs in absolute terms was 7% between the interferon-treated and the untreated patients. Sondak and Flaherty asked whether “this is the drug ... we were waiting for” in an editorial published in *The Lancet*^12^. They brought forward a very important point, arguing that the strongest interferon benefit was observed in the subset of melanoma patients with micrometastases in the sentinel node. Not only was an improvement in rfs evident for this patient population, but also in distant metastases-free survival (dmfs). A less favourable outcome for patients receiving pegylated interferon alfa 2b as compared with untreated controls was observed in the N2 patient population with clinically detectable macrometastases of the lymph nodes.

## Is There Any Evidence That Interferon-Treated Patients May Benefit More If They Have Micro- as Compared with Macrometastases of the Lymph Nodes?

I believe that there is enough evidence to support the hypothesis that interferon-treated patients may benefit more if they have micrometastases than if they have macrometastases of the lymph nodes. In two clinical trials on a total of more than 2500 patients (eortc 18952 and eortc 18991) published in recent years, a clear dependency of interferon responsiveness on tumour load (stage iib/c > iiia > iiib) was observed^11^,^13^. However, the biology behind those observations is currently unclear.

Furthermore, for the second time, melanoma patients with an ulcerated primary tumour treated with either conventional interferon alfa 2b or pegylated interferon alfa 2b were demonstrated to respond better to interferons than did patients with a non-ulcerated melanoma primary^11^. This phenomenon has not been well understood biologically till now. In addition, some support for these findings comes from the Wheatley *et al.*^3^ meta-analysis. Hence, ulceration of the primary tumour appears to be a predictive biologic parameter for responsiveness to interferon in the absence of any other validated molecular or immunologic markers for interferon response. The interesting observation of a correlation between the development of autoimmunity during interferon treatment and a better treatment outcome was consistently shown in two reports^14^,^15^. In an eortc study, however, those results could not be confirmed when only a seroconversion of auto-antibodies during treatment was considered to be an autoimmune event. In any case, autoimmunity is clearly not a predictive marker before treatment initiation, but more a surrogate marker of response.

The eortc Melanoma Group will soon initiate a prospective randomized phase iii trial on sentinel node–negative patients with an ulcerated primary melanoma of more than 1.0 mm tumour thickness. Pegylated interferon alfa 2b will be compared with observation alone in approximately 1000 melanoma patients. That study, as well as previous clinical trials, contains a translational research portion that will consider several potential biomarkers for interferon responsiveness.

## Have Any New Trial Results Emerged That May Contribute to Current Knowledge on the Adjuvant Treatment of Melanoma with Interferon?

In the January issue of the *Journal of Clinical Oncology,* Pectasides and coworkers from the Hellenic Cooperative Oncology Group published the results of a prospective randomized study on 4-week intravenous induction therapy with interferon alfa 2b as compared with a full year of a modified hdi 2b 16. A total of 364 patients were enrolled, of whom 353 were evaluable for the study endpoints. Approximately 30% of the treated patients were stage iib/c, and roughly 60 %were stage iiia/b/c, with most being stage iiib. In 11% of the patients in both treatment arms, the appropriate staging category was not known^16^.

Compared with the original report and current treatment recommendations on hdi, the Greek patients received a dose of only 15×10^6^ IU/m^2^ daily during the induction phase. This was a 25% reduction as compared with the doses used by Kirkwood *et al.* The maintenance dose was also modified from the approved dose (10×10^6^ IU/m^2^ thrice weekly, considered a 50% dose reduction) for “patients’ convenience of dosing and administration.” The statistical analysis was confined to the primary endpoint of rfs and secondary endpoints such as dmfs and os in the intent-to-treat population.

The authors said that the study design was based on the hypothesis that the 1-month induction regimen (arm A) would be considered as efficacious as the conventional regimen (arm B) if the relapse rate at 3 years from study entry were to be no more than 15% higher in arm A than in arm B. A sample size of 308 patients was required to achieve a power of 85% in a one-sided test at a significance level of 0.05. The target accrual was set at 342 patients (171 patients per arm), anticipating a 10% withdrawal rate. Only a few patients (3 in arm A and 10 in arm B) discontinued the study treatment because of toxicity. A dose reduction was required in 25% of all patients during the intravenous treatment phase and in 9.6% of arm B patients during the subcutaneous maintenance phase. No unexpected toxicities or treatment-related deaths occurred. At a median follow-up of 63 months, no differences in rfs, dmfs, or os have been observed between the two treatment arms^16^.

## Is This Clinical Trial Changing the Current Treatment Recommendations for Patients with High Risk of Melanoma Relapse?

I have some doubts about the conclusion by Pectasides *et al.*^16^ that 1 month of hdi 2b has the same clinical benefit as 1 year of hdi. The following pitfalls can be critically discussed:

- The study did not aim to use the originally described and subsequently approved dose of hdi used in the ecog trials^4^. A 25% dose reduction during the intravenous induction phase and a 50% dose reduction during the subcutaneous maintenance phase leads to a consideration that only “modified hdi” was tested by the Hellenic Cooperative Oncology Group.
- The statistical design of the Greek trial is inappropriate for a modern clinical trial. None of the previous clinical trials conducted with conventional interferons or pegylated interferon alfa 2b—nor the meta-analysis by Wheatley *et al.*—showed more than a 10% difference in dfs for one treatment arm as compared with another treatment arm or observation alone. Notably, most recent interferon alfa trials have compared one or two different interferon schemes with untreated controls. Because interferon has limited but clear activity in melanoma, expectation of a demonstration of a statistically significant difference between two interferon schedules appears to be very optimistic. It is much easier to show differences if interferon is compared with no treatment. The probability that 12 months of modified hdi is 15% better than 4 weeks of modified hdi is extremely low. In the trial that led to the approval of hdi for adjuvant treatment of melanoma, the absolute difference between hdi and observation alone was only 7%!

The Greek trial was therefore clearly too underpowered to detect small differences in efficacy between the two arms. In their article, Pectasides *et al.* spent more than half the discussion on a justification of their “non-inferiority study design.” They concluded that, according to the non-inferiority margin defined at study design, it can be ruled out that the 3-year relapse rate in arm A (1 month of treatment only) is higher by 15% or more than the relapse rate in arm B.

The authors correctly emphasize that “this study does not rule out the possibility of smaller differences between groups A and B.” They are referring to the ongoing ecog 1697 study comparing adjuvant hdi for 1 month with observation alone, which “will give us more insight on the efficacy of this regimen in the adjuvant setting.” Whether this outlook on the ecog 1697 trial is a fair one can be discussed, given that the ecog study is being performed in a different setting, mainly with patients who are sentinel node–negative and with only a few whose lymph nodes not clinically palpable. Furthermore, the ecog 1697 study uses the approved induction-phase dose of interferon alfa 2b (20×10^6^ IU daily). The ecog study will, in principle, be able to respond to the question of whether the use of a high-dose induction phase at all is superior to a “wait and see” policy.

## Is It Time to Switch from Conventional HDI to the Greek 4-Week Modified HDI Induction Treatment?

The Greek trial was truly underpowered: minor (<15%) but clinically meaningful differences in efficacy are not detectable. The regimen used was an unconventional and unapproved interferon alfa 2b scheme. On the other hand, a modified 4-week scheme seems to be attractive because of lesser toxicity and less time and fewer dollars consumed. This scheme could be considered for patients who are not willing to tolerate a classical 1-year hdi or who are not eligible for hdi for other reasons. In any case, the results of the complex Greek study need to be explained to patients, together with the various pros and cons.

Because the planning, conduct, and follow-up of a melanoma trial require approximately 10 years, it is very unlikely that this regimen will be tested again in another trial. Thus, we need to live with some open questions concerning the Greek trial.

## What Are the Future Perspectives?

Large clinical trials on 1000 or more intermediate- and high-risk melanoma patients are currently under evaluation or approaching initiation. The ecog 1697 trial on a 4-week hdi schedule has already been mentioned. A recently activated eortc trial sponsored by Bristol–Myers Squibb on adjuvant ipilimumab compared with an intravenous placebo is under way, but that trial will certainly need a couple of years before final results are published. A large eortc trial sponsored by GlaxoSmithKline on a mage-a3 (melanoma antigen family A, 3) vaccination will also be initiated in the second quarter of 2009. That trial is a good example of a newly developed targeted therapy, because only patients with mage-a3-positive melanoma will be considered.

A huge package of translational research objectives is attached to all the newly designed clinical trials in the adjuvant setting. Hopefully, melanoma treatment will soon be able to be individualized on the basis of new predictive tumour-tissue or blood biomarkers. Without such biomarkers, there is only a little hope that more than a small subset of melanoma patients will reap benefit from adjuvant treatment. Let us cross our fingers that gene expression profiling, together with sophisticated new techniques, is leading to more effective treatment modalities for melanoma patients.
